# Identification of Potential Key Genes for Pathogenesis and Prognosis in Prostate Cancer by Integrated Analysis of Gene Expression Profiles and the Cancer Genome Atlas

**DOI:** 10.3389/fonc.2020.00809

**Published:** 2020-06-01

**Authors:** Shuang Liu, Wenxin Wang, Yan Zhao, Kaige Liang, Yaojiang Huang

**Affiliations:** ^1^Beijing Engineering Research Center of Food Environment and Public Health, Minzu University of China, Beijing, China; ^2^Hospitial of Chifeng City, Chifeng, China; ^3^Xuzhou Central Hospital, Xuzhou, China; ^4^Department of Environmental Health, Harvard T. H. Chan School of Public Health, Boston, MA, United States

**Keywords:** prostate cancer, GEO, TCGA, bioinformatics, survival, biomarker

## Abstract

**Background:** Prostate cancer (PCa)is a malignancy of the urinary system with a high incidence, which is the second most common male cancer in the world. There are still huge challenges in the treatment of prostate cancer. It is urgent to screen out potential key biomarkers for the pathogenesis and prognosis of PCa.

**Methods:** Multiple gene differential expression profile datasets of PCa tissues and normal prostate tissues were integrated analysis by R software. Gene Ontology (GO) and Kyoto Encyclopedia of Genes and Genomes (KEGG) pathway analysis of the overlapping Differentially Expressed Genes (DEG) were performed. The STRING online database was used in conjunction with Cytospace software for protein-protein interaction (PPI) network analysis to define hub genes. The relative mRNA expression of hub genes was detected in Gene Expression Profiling Interactive Analysis (GEPIA) database. A prognostic gene signature was identified by Univariate and multivariate Cox regression analysis.

**Results:** Three hundred twelve up-regulated genes and 85 down-regulated genes were identified from three gene expression profiles (GSE69223, GSE3325, GSE55945) and The Cancer Genome Atlas Prostate Adenocarcinoma (TCGA-PRAD) dataset. Seven hub genes (FGF2, FLNA, FLNC, VCL, CAV1, ACTC1, and MYLK) further were detected, which related to the pathogenesis of PCa. Seven prognostic genes (BCO1, BAIAP2L2, C7, AP000844.2, ASB9, MKI67P1, and TMEM272) were screened to construct a prognostic gene signature, which shows good predictive power for survival by the ROC curve analysis.

**Conclusions:** We identified a robust set of new potential key genes in PCa, which would provide reliable biomarkers for early diagnosis and prognosis and would promote molecular targeting therapy for PCa.

## Introduction

Prostate cancer (PCa) is a global public problem threatening human health and life, which is harmful to the male genitourinary system (1). According to the American Cancer Association and the National Cancer Institute in 2019, PCa is one of the most common malignancy, with about 3,650,030 columns (2). In the latest research, the incidence and mortality increased year by year on the incidence and mortality of PCa, accounting for more than 90% of reproductive organ cancer diseases. In China, although the incidence of PCa is slightly lower than that in European and American countries, the incidence of PCa is increasing year by year with the aggravation of population aging and changes in dietary structure and living environment (3).

At present, traditional prostate biopsy brings great pain to patients. The most commonly used Prostate cancer biomarker for screening prostate cancer in clinic is serum prostate specific antigen (PSA), which has low specificity and great limitations. It is extremely urgent to screen new early diagnosis and prognostic markers by bioinformatics for pathogenesis and prognosis of prostate cancer.

Advances in microarrays and high-throughput sequencing technologies play an important role in the study of the occurrence and development of cancer and provide effective tools, and have been widely used in screening biomarkers for cancer diagnosis, treatment and prognosis. At the same time, in order to change the limitation of different technology platforms or small sample size, the integrated bioinformatics method is used in cancer research. The combined application of multiple databases can integrate data from different independent studies and obtain more clinical samples for data mining, so as to achieve more robust and accurate analysis. These new bioinformatics technologies have provided tremendous potential for the advancement of cancer biomarkers research.

In our study, our aim was to screen potential key genes related to the pathogenesis and good prognostic markers through integrated bioinformatics analysis. For this purpose, we first screened overlapping differentially expressed genes (DEGs) from multiple microarrays and TCGA PRAD RNA-seq data. Gene Ontology (GO) function and Kyoto Encyclopedia of Genes and Genomes (KEGG) pathway analysis for the overlapping differentially expressed genes was annotated. Finally, the potential key genes for pathogenesis and prognosis in PCa were identified by using PPI network and survival analysis.

## Materials and Methods

### Data Downloading

The GEO database was used to download the raw datasets for comparing gene expression between PCa tissues and normal tissues. The gene expression data of GSE69223, GSE3325, and GSE55945 were download based on the platform of GPL570 (Affymetrix Human Genome U133 Plus 2.0 Array). The pretreatment of Level 3 mRNA expression data information was downloaded from the TCGA database. And clinical samples related to prostate cancer were selected. This dataset included 499 samples of prostate cancer, 52 samples of normal and clinical information of these corresponding samples.

### Data Preprocessing and Differentially Expressed Genes (DEGs) Screening

For the raw expression data of each GEO dataset, Robust multi-array average (RMA) was used to correct and normalize the background. The merging of three GEO datasets was accomplished by using perl language, data information was not changed. The SVA package of R soft was eliminated batch effects and other unrelated variables in high-throughput experiments (4). For the combined GEO data, the k-Nearest Neighbor method (KNN) complements the missing values. The DEGs of PCa and normal tissues were detected by limma package of R software (5), *P*-Val < 0.05 and absolute logFC > 1. Using edgeR package of R software to normalize TCGA datasets and screen DEGs (6), *P*-Val < 0.05 and absolute logFC >1. The Venn diagram of the overlapping DEGs were outputted by Funrich software (Funrich 3.1.3).

### Enrichment Analysis of Gene Ontology (GO) and Kyoto Encyclopedia of Genes and Genomes (KEGG) Pathway

ClusterProfiler package (7) performed gene ontology (GO) enrichment analysis (3) and Kyoto Encyclopedia of Genes and Genomes (KEGG) pathway analysis (8) for the overlapping DEGs. GO enrichment analysis mainly described the biological processes (BP), cellular components (CC) and molecular functions (MF) correlated with differentially expressed genes. KEGG pathway analysis revealed biological pathways associated with DEGs. adjust *P*-value < 0.05 were used as the cutoff standard.

### Construction of Protein-Protein Interaction (PPI) Network and Modules Selection

In order to research the interactions among the overlapping DEGs, PPI network was identified by the STRING online database (http://string-db.org) (9). The interaction with a confidence score of ≥ 0.4 was considered significant and retained. R software was applied to further analyze the degree of calculation and draw a ranking chart based on the degree. PPI network's information was further imported into Cytoscape software (10) for subsequent analysis. We used Molecular Complex Detection (MCODE) app in Cytoscape for module analysis to detect cluster modules (11), Parameter setting:degree cut off:2, node score cut off:0.2, k-core cut off:2, and max. depth cut off :100. We applied ClusterProfiler to perform GO enrichment analysis on the two modules. And Biological pathway analysis was done by Funrich 3.1.3.

### Verification of Hub Gene in GEPIA Database

Gene Expression Profiling Interactive Analysis (GEPIA, http://gepia.cancer.pku.cn/) database is a web server based on data from the UCSC Xena program. The functions of the database are divided into two main themes: expression analysis and custom data analysis, which can be used to analyze gene expression differences between various cancers and normal tissues, and overall survival rate, etc. GEPIA database can be used to analyze differences in expression between cancer and normal tissues as well as overall survival (12). We further validated the mRNA expression level of the hub gene through the GEPIA database. The critical condition was set to |Log2FC| Cutoff:2, *p*-value Cutoff:0.05.

### Survival Analysis

In the TCGA database, Clinical data and clinical related information was downloaded, and then we need to obtain Over Survival (OS) data, excluding entries for cases without data. The remaining case data was used for further survival analysis (13–15). Potential genes highly associated with overall survival were recognized based on univariate Cox proportional hazard regression analysis. Cox proportional hazard regression analysis screened prognostic gene signature from the DEGs, *P* < 0.05 (16). The Cox proportional hazard regression model was constructed with prognostic key genes as dependent variables for the purpose of estimate prognostic key genes' relative contribution to patient survival prediction. We constructed a predictive formula for gene characteristics and the following formula for this model is that risk score = expression of gene 1 × β 1 gene 1 + expression of gene 2 × β 2 gene 2 +…expression of gene n × β n gene n. The formula is a linear combination, of which gene expression values and regression coefficients (β) obtained from the multivariate Cox proportional risk regression model for each gene. According to the median risk score, the prostate cancer patients were divided into high-risk group and low-risk group. Survival analysis of high-risk and low-risk groups was carried out with R packages-survival and survminer. In order to analyze the accuracy of survival prediction performance by using the risk score model, SurvivalROC package was used to construct the time-dependent receiver operating characteristic (ROC) curve. The prognostic gene signature's ability to predict clinical outcomes was determined by the area of the curve of AUC. When AUC > 0.5, the closer AUC is to 1, the prognosis was better. In addition, a comprehensive survival analysis was conducted based on the risk scoring model and clinical data (age, T stage and N stage) of prostate cancer.

### Statistical Analysis

The univariate and multivariate Cox proportional hazards regression analyses were conducted by the survival package of R software. Hazard ratio (HR) and 95% confidence interval (CI) were calculated to identify protective genes (HR < 1) or risk genes (HR > 1). The survival of high-risk patients and low-risk patients was analyzed using the Kaplan–Meier method.

## Results

**Figure 1:**
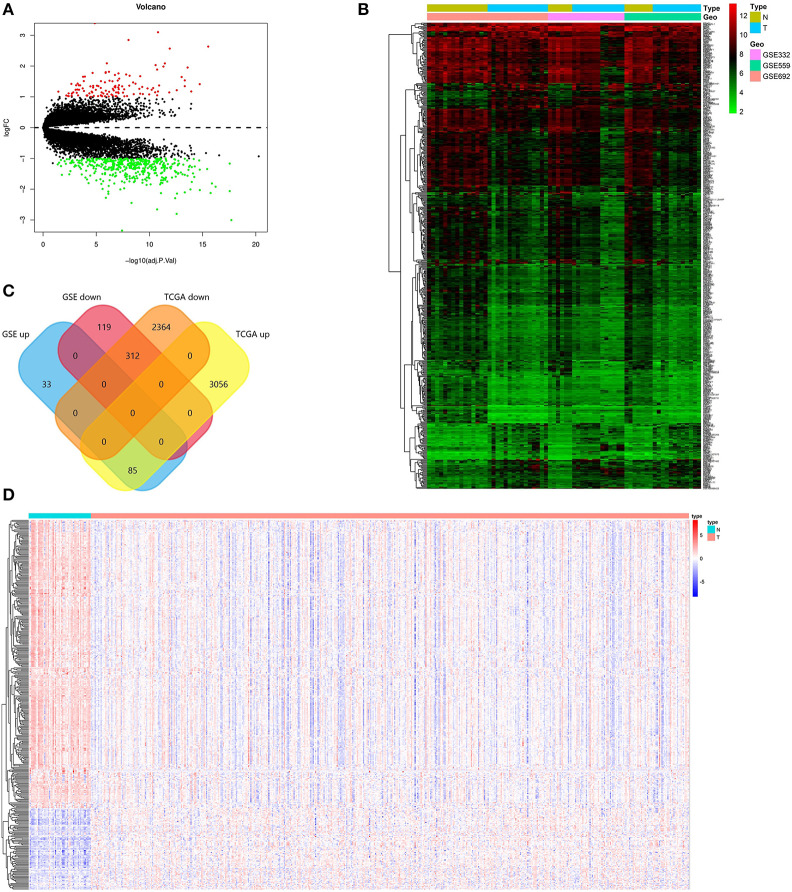
Identification of the DEGs. **(A)** Volcano plot of the integrated microarray of GSE69223, GSE3325, and GSE55945. The red nodes represent up-regulated DEGs. The green node indicates down-regulated DEGs. **(B)** A heatmap of all DEGs of the integrated microarray. Each column represents one dataset and each row represents one gene. The color changing from green to red represents the changing from downregulation to upregulation for the expression. **(C)** Venn diagrams of the overlapping DEGs between the integrated three microarrays and the TCGA PRAD dataset. **(D)** A heatmap of the overlapping DEGs in the TCGA PRAD dataset.

Screening criteria based on *P* < 0.01 and absolute log (FC) > 2, DEGs were screened between PCa and normal tissues in GSE69223, GSE3325, and GSE55945. Table 1 shows the detailed information about three GEO datasets. 547 DEGs were obtained, including 118 up-regulated genes and 429 down-regulated genes (Figures 1A,B, Supplementary Table 1). In the TCGA PRAD dataset, 5,817 DEGs were screened [*p* < 0.01 and |log(FC)| >1], which consisted of 3,141 up-regulated genes and 2,676 down-regulated genes (Supplementary Table 2). At the data of GSE69223, GSE3325, GSE55945, and TCGA PRAD dataset, 397 overlapping differentially expressed genes were found. Among them, 312 up-regulated genes and 85 down-regulated genes (Figure 1C). The overlapping up-regulated DEGs and the overlapping down-regulated DEGs were summarized in (Figure 1D; Tables 2, 3).

**Table 1:** Information of the three GEO datasets.

**Dataset**	**References**	**Platform**	**Tumor**	**Normal**
GSE69223	Meller et al. (17)	[HG-U133_Plus_2] Affymetrix Human Genome U133 Plus 2.0 Array	15	15
GSE3325	Varambally et al. (18)	[HG-U133_Plus_2] Affymetrix Human Genome U133 Plus 2.0 Array	13	6
GSE55945	Arredouani et al. (19)	[HG-U133_Plus_2] Affymetrix Human Genome U133 Plus 2.0 Array	13	8

**Table 2:** The overlapping upregulated DEGs between GSE69223, GSE3325, GSE55945, and TCGA PRAD dataset.

**Upregulated DEGs names**
HPN,FRMPD3,FOXD3-AS1,DNAH5,PYCR1,POPDC3,DBNDD1,PCA3,DUS1L,
STX19,DLX1, HOXC4,HOXC6,ELL3,HIST1,H2AM,SMIM22,MARCKSL1,
SLC7A11,APOF,RAB17,LUZP2,CGREF1,SIM2,EPCAM,TLCD1,TDRD1,FEV,
OR51E2,MS4A8,FAM222A,TGM3,AMACR,MELK,NEK5,ACSM1,THBS4,B3GAT1,
DLX6-AS1,GCNT1,ADAM2,GDF15,TRPM4,FOXD1,PPM1E,NKAIN1,PRAC1,
TWIST1,BICD1,CTHRC1,GJB1,FFAR2,ZIC2,ATP8A2,PCAT18,TMEM178A,
ELAVL2,KCNH8,PTPRT,VSTM2L,HMMR,HIST1H2AE,MYO6,GPR160,
TOX3,BUB1B,INSM1,C12orf56,MMP26,RRM2,NETO2,EZH2,SDK1,TFF3,ERG,
C15orf48,TMEM45B,PLA2G7,FOLH1B,ABCC4,COL2A1,DNASE2B,PRR16,
FABP5,MIPEP,OR51E1

**Table 3:** The overlapping downregulated DEGs between GSE69223, GSE3325, GSE55945, and TCGA PRAD dataset.

**Downregulated DEGs names**
TSLP,NUDT10,KCNJ3,FAT4,RAB40A,SBSPON,CCND2,GLIS3,ITIH5,RBMS3,
KCNAB1,ATRNL1,HOXD13,SPOCK3,NRK,SLC8A1,TBX4,SPON1,JAZF1,SGCB,
C1QTNF7,TWIST2,AFAP1L2,ANGPTL1,RNF180,HS3ST3A1,PLAC9,DAAM2,
SNAI2,NELL2COL4A6,C2orf88,VCL,CFD,ME1,NHS,SEMA5A,AJUBA,OLFML1,
CCDC8,PEG3,MAMDC2,ANGPT1,RBP4,FAM162B,C8orf88,RNF175,FBXO17,
FERMT2,MAP1B,PLCL1,TRPC1,ANO5,TIMP3,PENK,ASPA,FXYD6,CLIC6,
CCDC136,GSTM5,NT5E,C5orf34,EDNRB,PTGS1,LINGO2,PHYHIP,NDNF,FGF2,
ZNF516,DKK3,CLIP4,BMP5,CCDC80,PCDH9,DCN,LPAR1,SLMAP,PRICKLE2,
TPM2,MSRB3,SRD5A2,EID3,RNF112,ADAMTS5,ITGB1BP2,PALLD,CLU,
GPM6B,MAMLD1,CAV2,LRRN3,TRIM6,PCDH10,ITGA1,RASL12,TSHZ3,
DAB1,RND3,LGR6,EFEMP1,DUOXA1,LMOD1,TCEAL2,PTGIS,ZNF423,GSTM3,
TCF7L1,ID4,FZD7,SCARA3,CAV1,SCN7A,SGCA,MYLK,TMEM200B,ACSS3,
HCG11,RAB9B,SMOC1,RGS7BP,AHNAK2,TIMP4,ATP1A2,SLC2A5,DNAJB4,
TSPAN18,S100A6,FBXL22,ST8SIA1,DZIP1,CDC42EP3,LINC01082,VWA5A,
MLC1,RGS9,MXRA7,PTGER2,PNMA1,NDP,PLBD1,TGFBR3,TBX5,FHOD3,
STARD4,FRMD6,PGM5,HLF,POPDC2,STAC,GSTM1,EFEMP2,MBNL1AS1,
MYOCD,RBFOX3,FAM107A,CFL2,DNAJB5,BDNF,AOX1,ANO4,EPHA7,SLC14A1,
RBPMS2,SYNC,SLC16A5,FLNA,DDR2,C3orf70,INMT,MRVI1,DOCK3,
PPP1R14A,CCDC69,HSPB8,PPP1R1A,TMEM47,DPT,GPR87,TCEAL7,SGCG,
SNX7,KLHL13,TRHDE,CYP3A5,GJA1,MAL,WFDC1,FLNC,B3GALT2,FOXF2,
RRAS,CPNE5,MEIS1,CXCL13,PABPC4L,IGSF1,FILIP1L,MYOF,EPB41L3,
GPRASP1,VIT,PDGFC,C2orf40,DSC3,C12orf75,EPHA2,GPX8,SLC24A3,
CD200,C7,CRYAB,ZNF204P,SLC47A1,SCGB3A1,EPHB1,RCAN2,FHL1,ASB2,
SLITRK6,CHRDL1,BCL11A,IL33,PRIMA1,GATA3,NEXN,SNHG18,PPP1R3C,
LSAMP,UBXN10AS1,TMEM158,TENM2,PTGDS,FOXQ1,GPM6A,FOXF1,TGFB1I1,
ACOX2,PARM1,HOXD10,RGN,KRT23,SYNDIG1,PGMAS1,WIF1,HSPB6,KCNJ8,
HSPB3,EFS,PRTFDC1,SCGB1A1,AOC3,GSTP1,ZNF536,EDNRA,MIR100HG,
SOWAHA,GSTM2,NSG1,MYH11,ROR2,SMR3B,NEURL1B,LINC00844,
TMEM100,ID3,ACTC1,LAMB3,OGN,MYL9,LRCH2,IER3,SERPINF1,SYNPO2,
DDIT4L,KIAA1210,PLN,PDZRN4,HSD11B1,IGDCC4,MEIS2,PYGM,PLA2G4A,
DKK1,IP6K3,SMTNL2,SLC18A2,KRT14,CNN1,MYH6,JAKMIP1,CCK,ID1,
KRT5,FLRT3,MME,SNX31,CYP4B1,LINC00261,MIR205HG,SLC39A2,UPK1A,
BEX1,PTGS2,KRT13,TRG-AS1,TGM4,CD177,NEFH,SERPINB11

### Functional and Pathway Enrichment Analysis of the Overlapping DEGs

**Figure 2:**
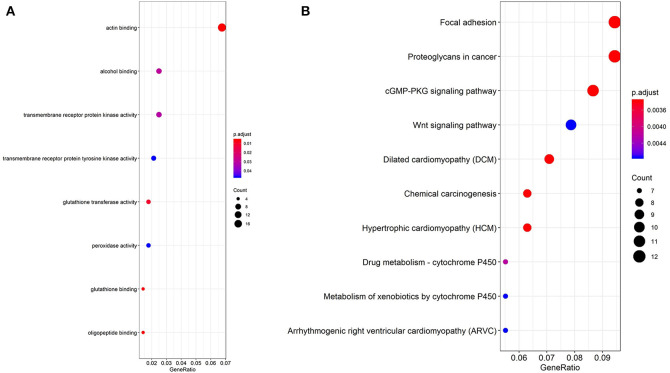
GO analysis and KEGG pathway analysis of the overlapping DEGs. **(A)** GO enrichment analyses of the overlapping DEGs. **(B)** KEGG pathway enrichment analysis of the overlapping DEGs.

In order to explore the enrichment of the functions and pathways of the overlapping DEGs, Gene Ontology and Kyoto Encyclopedia of Genes and Genomes pathway analysis were performed. Gene Ontology analysis showed that the overlapping DEGs were mainly enriched in actin binding, transmembrane receptor protein kinase activity, alcohol binding, transmembrane receptor protein tyrosine kinase activity (Figure 2A, Supplementary Table 3). The pathway enrichment analysis showed that the overlapping DEGs were associated with pathways such as Focal adhesion, Proteoglycans in cancer, cGMP-PKG signaling pathway, Wnt signaling pathway and PI3K-Akt signaling pathway (Figure 2B, Supplementary Table 4).

### PPI Network Construction and Hub Gene Identification

**Figure 3:**
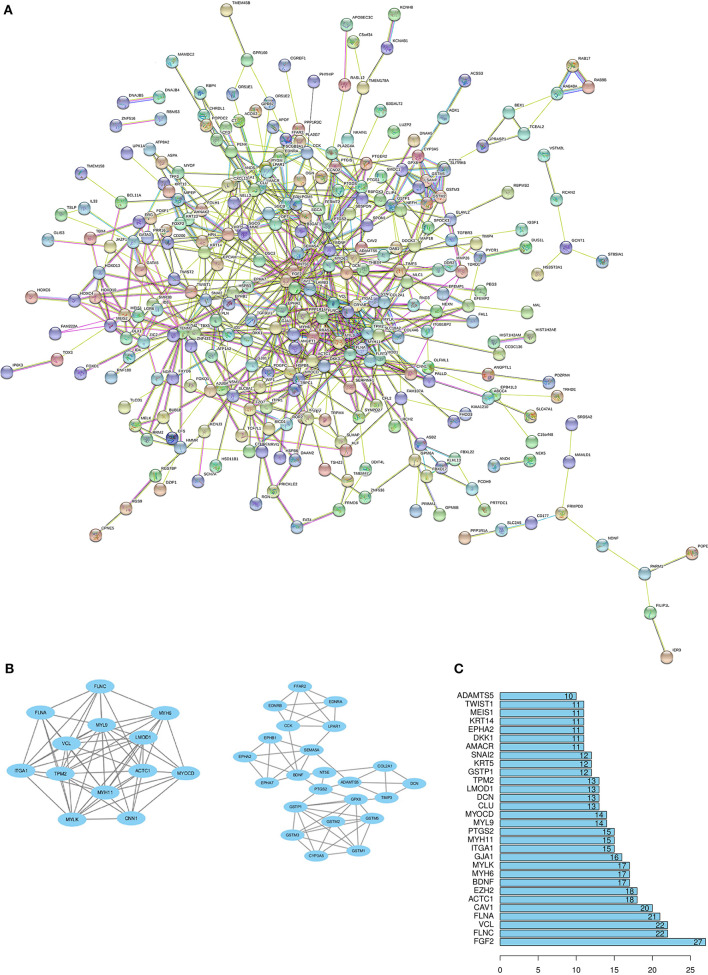
Protein–protein interactions (PPI) network, module analysis, and hub gene identification. **(A)** Using the STRING online database to construct PPI network of the overlapping DEGs. **(B)** The degree of protein interaction ranks to determine 10 hub genes in Protein-Protein Interaction. **(C)** TWO modules were screened by using MCODE app in Cytoscape software. The top module score is 10, another module score is 5.18.

**Figure 4:**
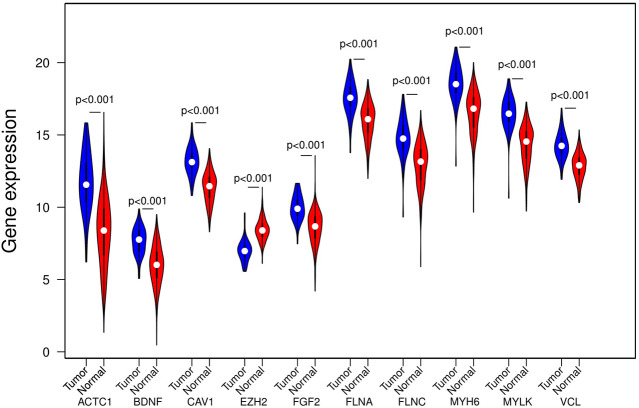
Expression of the ten hub DEGs in tumor and normal tissues in TCGA PRAD dataset. Expression values of the ten hub DEGs are log 2 -transformed.

**Figure 5:**
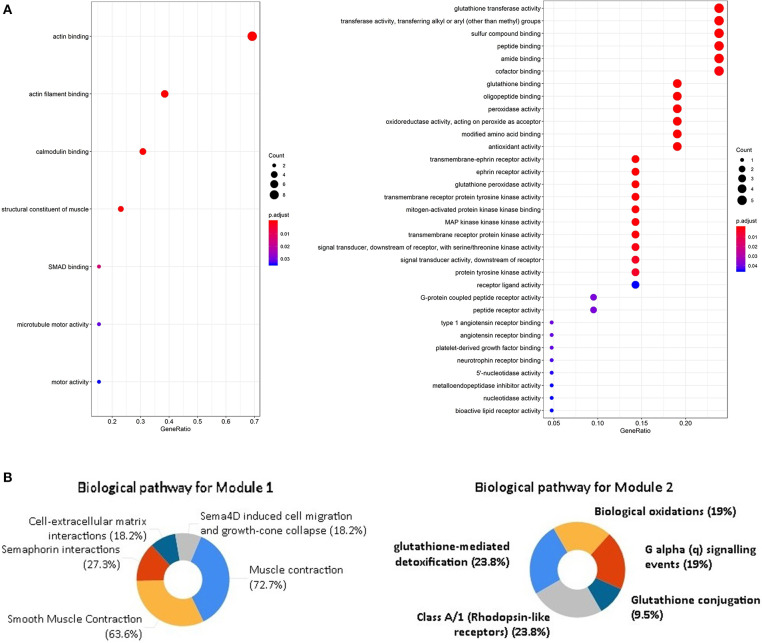
GO analysis and biological pathway enrichment analyses of the module. **(A)** GO analysis of the DEGs in two modules. The y-axis labels represent clustered GO terms. the x-axis shows the ratio of the number of genes enriched in one GO term to the number of genes in two modules. **(B)** Biological pathway enrichment analysis of the DEGs in two modules.

Through the SRTING online database, we constructed a protein-protein interaction network for the overlapping DEGs, which included 381 nodes and 675 edges (Figure 3A). Using R software, 10 genes with the highest connectivity were screened as FGF2, FLNA, VCL, FLNC, CAV1, ACTC1, EZH2, BDNF, MYH6, and MYLK (Figure 3B). Based on the TCGA PRAD dataset, we mapped the expression of 10 hub genes in PCa and normal tissues (Figure 4). Based on the MCODE in Cytospace software, the two gene by using the internal relationship among network proteins. The most important cluster consists of 13 nodes and 60 edges. Another cluster consists of 23 nodes and 57 edges (Figure 3C). GO analysis results showed that the cluster 1 was closely correlated with mitotic nuclear division, cell division, mitotic cytokinesis, midbody, centrosome, and nucleus (Figure 5A, Supplementary Table 5). The functions associated with the Cluster 2 are glutathione transferase activity, glutathione binding, oligopeptide binding, transferase activity, transferring alkyl or aryl (other than methyl) groups, peroxidase activity (Figure 5A, Supplementary Table 6). The results of pathway enrichment analysis, the cluster 1 was mainly enriched in muscle contraction, smooth muscle, contraction, semaphorin interactions, cell-extracellular matrix interaction, sema4D induced cell migration and growth-cone collaose (Figure 5B). Another cluster was mainly related to biological pathways, including glutathione-mediated detoxification, class A/1(Rhodopsin-like receptors), Biological oxidations.

### Application of GEPIA Databases to Verify the Expression Level of the Hub Gene

**Figure 6:**
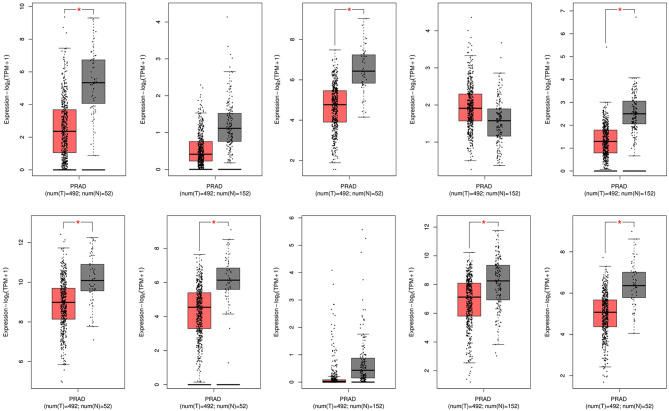
mRNA expression levels of 10 hub genes in the GEPIA database. PRAD, Prostate adenocarcinoma.

In the GEPIA database, differences in transcriptional expression of the hub gene between PCa tissues and normal tissues were again verified. Combining with the box plot results, seven potential key genes further were screened out. Based on the GEPIA database to test the relative expression of hub gene mRNA, it was determined that FGF2, FLNC, VCL, FLNA, CAV1, ACTC1, MYLK may be closely related to the occurrence and development of PCa (Figure 6).

### Survival Analysis

**Figure 7:**
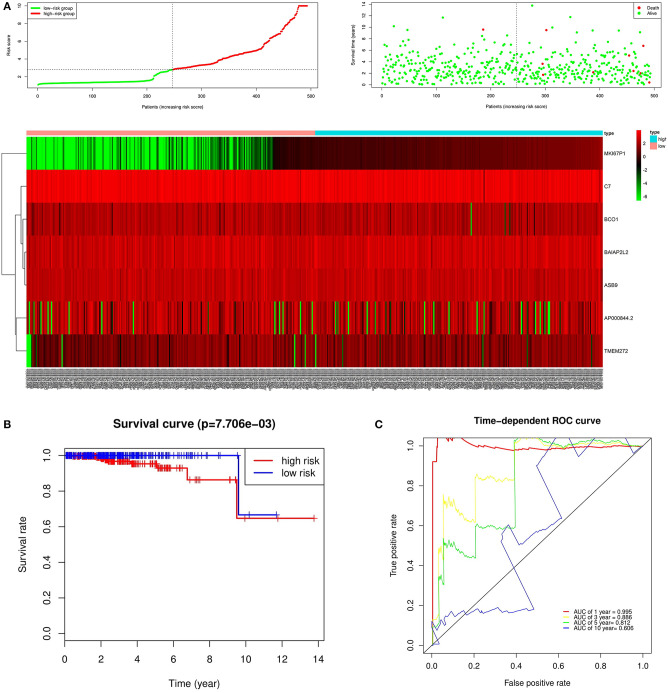
Prognostic analysis of seven genes in the patients in TCGA PRAD dataset. **(A)** The first figure shows the distribution of risk scores in low-risk group and high-risk group. The second figure shows the distribution of survival status of patients in low-risk group and high-risk group. the green point represents alive, and the red point represents death. The third figure shows a heatmap of seven prognostic genes **(B)** The survival curves for low- and high-risk groups (*p* = 7.706e-03). **(C)** ROC curve for predicting prognosis gene performance based on risk score.

**Figure 8:**
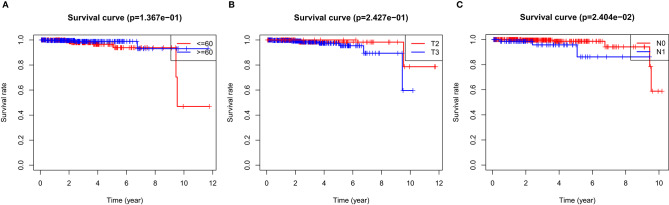
OS survival was analyzed by risk score and clinical data stratification of prostate cancer. Stratified analysis was conducted from the following clinical parameters: **(A)** Age of PCa patients; **(B)** T stage of tumor; **(C)** N stage of tumor Tables.

In the over survival (OS) data obtained from TCGA database, we found that the longest follow-up time was 5,024 days, the shortest follow-up time was 23 days, and the average follow-up time was 1,088 days. Through univariate Cox proportional hazard regression model, a total of 38 genes significantly related to survival time were identified (*P* < 0.05) (Supplementary Table 7). Using multivariate Cox proportional hazard regression model, seven genes were screened: BCO1, BAIAP2L2, C7, AP000844.2, ASB9, MKI67P1, TMEM272. These seven genes constituted a prognostic signature for PCa. Among these seven genes, HR < 1 were identified as a protective prognostic gene including C7 and BAIAP2L2, while HR > 1 were identified as a risk prognostic gene, including BCO1, AP000844.2, ASB9, MKI67P1, and TMEM272 (Table 4, Supplementary Table 8). According to the median risk score, a total of 246 patients with a risk score greater than the median risk score was divided into the high-risk group, while the other 247 patients were divided into the low-risk group. According to the median risk score, 246 patients with a risk score greater than the median risk score were in the high-risk group, and 247 patients with a risk score less than the median risk score were assigned to the low-risk group. The risk score of the TCGA PRAD dataset was showed in Figure 7A. In comparison, the survival rate of high-risk group and low-risk group declined with the passage of time (Figure 7B). The difference in 10-year survival rate between the high risk group and the low risk group is small, it may be due to the high survival rate of prostate cancer patients compared with other cancers. In the high-risk group, the OS rates at 1, 3, 5, and 10 years were 98.30% (95% CI = 96.40–100%), 95.30% (95% CI = 91.50–99.30%), 93% (95% CI = 87.1–99.10%), and 64.7% (95%Cl = 36–100%). In the low-risk group, the OS rates were 100% for1, 3 and 5 years, the OS rates at 10 years were 66.7% (95% CI = 30–100%). The AUC was 0.995, 0.886, 0.812, and 0.606 for 1, 3, 5, and 10 years, the results show that the prognosis gene signature shows good survival prediction ability (Figure 7C). Analysis of the seven-gene signature and analysis of age, T stage and N stage of prostate cancer patients also showed a high predictive value for overall survival of prostate cancer patients (*P* < 0.001) (Figure 8).

**Table 4:** Prognostic value of the seven genes in the PCa patients of the TCGA cohort.

**Gene symbol**	**Univariate analysis**		**Multivariate analysis**		
	**Hazard ratio (95% CI)**	***p*-value**	**Hazard ratio (95% CI)**	***p*-value**	**Coefficient**
BCO1	2.619(1.438–4.768)	0.002	1.023(1.006–1.040)	0.007	0.022
BAIAP2L2	1.644(1.187–2.278)	0.003	0.999 (0.997–1.000)	0.037	−0.002
C7	0.657(0.491–0.879)	0.005	0.999 (0.999–1.000)	0.074	−0.001
AP000844.2	1.743(1.2151–2.499)	0.003	1.004 (1.002–1.004)	<0.001	0.004
ASB9	1.828(1.315–2.541)	<0.001	1.002(1.002–1.006)	0.039	0.001
MKI67P1	5.033(1.977–12.804)	0.001	2.288(1.307–4.005)	0.004	0.828
TMEM272	2.545(1.479–4.380)	0.001	1.05(0.989–1.123)	0.106	0.053

## Discussion

We identified differentially expressed genes between PCa tissues and normal tissues based on gene expression profiles and TCGA high-throughput RNA-seq dataset, including 312 up-regulated genes and 85 down-regulated genes in this study. The overlapping DEGs participated in actin binding, transmembrane receptor protein kinase activity, alcohol binding. For KEGG pathway analysis, the pathways related to the overlapping DEGs are Focal adhesion, Proteoglycans in cancer, cGMP-PKG signaling pathway, Wnt signaling pathway and PI3K-Akt signaling pathway. CGMP-PKG/Wnt/PI3K-Akt signaling pathway are important gene signaling pathways in carcinogenesis, which are closely related to tumor growth, cancer proliferation, apoptosis and cell carcinogenesis. Through the application of bioinformatics technology, we identified the potential key genes associated with the occurrence and development of prostate cancer as FGF2, FLNA, VCL, FLNC, CAV1, ACTC1, and MYLK. Potential key genes can help identify new molecules or pathways that may be involved in the diagnosis and treatment of prostate cancer, and provide new insights into its pathogenesis.

Fibroblast growth factor 2 (FGF2) was identified as one of the central genes with the highest degree of connectivity. Fibroblast growth factor 2 is a prototype member of the fibroblast growth factor family and interacts with its receptors to mediate receptor dimerization, phosphorylation and activation of signal transduction pathways. Fibroblast growth factor 2 signal is an imbalance in cancer cells and a key tumor promoter in the tumor microenvironment. Filamin A (FLNA) was reported to be necessary to regulate cell migration and invasion (20). More and more evidences show that it can also occur in different tumorigenesis processes, such as DNA damage and angiogenesis (21). Down regulation of the gene has been observed in a wide range of human malignant tumors, including gastric and renal cancer. However, some studies have reported that FLNA was significantly up-regulated in cervical cancer and has a good predictive effect, which can be used as a prognostic signature of cervical cancer (22). VCL encodes focal adhesion protein, participates in the formation of cytoskeleton and focal adhesion, and the gene also connects cells with extracellular matrix. VCL played an important role in cell adhesion, growth and proliferation, apoptosis, tumorigenesis and invasion (23). VCL may be a key protein marker and pathway related to bladder cancer by mass spectrometry and bioinformatics analysis (24). Filamin C (FLNC) is a member of actin-binding and cross-linked filament protein family, which promotes the migration and invasion of cancer cells (25). Some studies have shown that FLNC protein may be a target molecule for invasion and metastasis of hepatocellular carcinoma by iTRAQ technology (26). In gastric cancer, FLNC of GC cell line was down-regulated compared with normal tissue (27). Currently, quantitative proteomics has identified VCL and FLNC as two potential prognostic biomarkers and therapeutic targets for prostate cancer cell migration (28). Caveolin-1 (CAV1) is a carcinogenic membrane protein associated with endocytosis, extracellular matrix tissue, cholesterol distribution, cell migration and signal transduction (29). Some studies have found that CAV1 is involved in liver cancer, colon cancer, breast cancer, kidney cancer, lung cancer and other cancers, and acted as a promoter or inhibitor of cancer according to the type and stage of cancer (30). Alpha myocardial 1 (ACTC1) was a new independent prognostic and invasive marker in glioblastoma (GBM) (31). A series of experiments have proved that ACTC1 is a marker of invasion and prognosis of glioma. Previous studies have shown that ACTC1 can promote the proliferation and migration of breast cancer cells by proteomic assay (32). Myosin light chain kinase (MYLK) catalyzes the phosphorylation of myosin light chain and regulates the invasion and metastasis of some malignant tumors. MYLK promotes the progression of hepatocellular carcinoma by altering the cytoskeleton to enhance epithelial-mesenchymal transition (EMT) (33). In similar articles, the expression of MYLK in non-small cell lung cancer tissues was significantly lower than that in adjacent tissues and normal tissues by bioinformatics analysis (34).

We used univariate and multivariate Cox regression analysis to identify seven prognostic signatures for prostate cancer patients, including BCO1, BAIAP2L2, C7, AP000844.2, ASB9, MKI67P1, TMEM272. Seven prognostic signatures have good predictive value for prostate cancer patients. Complement C7 (C7) is a 121 kDa serum single-chain glycoprotein encoded by the C7 gene, and together with complement components C5, C6, C8, and C9 constitutes membrane attack complex (MAC). In cancer, a decrease in C7 expression levels in ovarian cancer patients is associated with worsening disease, but no prognostic value of C7 in ovarian cancer has been found. In the study of lung tumors, the continuous decline of C7 mRNA expression was observed and the clinical stage of lung tumor patients was correlated with the expression level of C7, and the prognosis was poor (35). In contrast, *in situ* hybridization and semi-quantitative reverse transcription polymerase chain reaction (RT-PCR) confirmed that C7 mRNA decreased or even disappeared in esophageal tumor cells (36). In our study, this gene was considered a protective prognosis gene, and these studies indicated that the complexity of the gene requires a lot of validation. Another protective prognostic gene is BAI1-associated protein 2-like 2 (BAIAP2L2), a member of the I-BAR family. BAIAP2L2 was up-regulated in lung adenocarcinoma tissues and various lung cancer cell lines, and overexpression of BAIAP2L2 can promote the proliferation and growth of lung adenocarcinoma cells (37). This gene had not been reported for prostate cancer related mechanisms.

Five risk prognostic genes identified in our study were BCO1, AP000844.2, ASB9, MKI67P1, TMEM272. Ankyrin repeat and SOCS box-containing 9 (ASB9) is a member of the ASB protein family, which is involved in the ubiquitination-mediated proteolytic pathway and in the regulation of cytokine signaling. Studies have reported that the expression of mRNA in ASB9 in CRC tissues is higher than that in corresponding normal tissues, and it can be used as an independent prognostic factor for colorectal cancer (38). In this study, ASB9 is highly expressed in prostate cancer, but the mechanism is still unclear. The enzyme β-carotene oxygenase 1 (BCO1) is a carotenoid lyase that was reported to be associated with a high consumption of tomato and tomato products rich in lycopene. BCO1 can cleave lycopene to produce carotenoids (39). However, there was also evidence that BCO1 is stably expressed in NB cells, and the expression of this gene inhibits the self-renewal and labeling of neuroblastoma CSC by regulating related miRNA (40). As well as the metastatic potential and enzyme activity of NB cells, BCO1 had a chemotherapeutic effect on malignant NB. AP000844.2, MKI67P1, and TMEM272 were rarely reported in current studies related to cancer, but were classified as risk prognostic genes in this study. These three genes will play a role in potential prognostic biomarker studies.

There are some shortcomings in this research:(1) Our research results were entirely based on the mining of public databases for bioinformatics analysis, so experiments are needed to verify the reliability of the results. (2) Our research was limited to the selection of candidate biomarker associated with the pathogenesis and Prognosis, which may lead to the neglect of some information. (3) Our data were from public databases, and the quality of the data has not been assessed.

## Conclusion

In summary, we identified seven genes that may be associated with the pathogenesis of PCa by using multiple gene expression profiles and TCGA PRAD dataset, two of which have been identified. We constructed a prognostic gene signature marker to predict the 1-, 3-, 5-, and 10-year survival rates of prostate cancer patients. Our results revealed potential key genes involved in the pathogenesis and prognosis of PCa and then, we provided a new method for risk stratification and prognosis prediction in prostate cancer patients. However, our research has been completed using data analysis from public databases, further experimental studies are urgently needed to verify our results.

## Data Availability Statement

Publicly available datasets were analyzed in this study. This data can be found here: http://www.ncbi.nlm.nih.gov/geo/ and http://cancergenome.nih.gov/.

## Author Contributions

SL, WW, YZ, and YH collected, analyzed, interpreted the data, and drafted the manuscript. YH conception, design, and had full access to all the data in the study and takes responsibility for the integrity of the data and the accuracy of the data analysis. KL participated in revising the manuscript. All authors read and approved the final version of the manuscript.

## Conflict of Interest

The authors declare that the research was conducted in the absence of any commercial or financial relationships that could be construed as a potential conflict of interest.

## Abbreviations

PCa: Prostate cancer
PRAD, Prostate Adenocarcinoma, GEO: Gene Expression Omnibus
TCGA: The Cancer Genome Atlas
GO: Gene Ontology
KEGG: Kyoto Encyclopedia of Genes and Genomes
RMA: Robust Multi-array Average
KNN: k-Nearest Neighbor method
DEG: Differentially Expressed Gene
PPI: Protein-Protein Interaction
MCODE: Molecular Complex Detection
GEPIA: Gene Expression Profiling Interactive Analys
ROC curve: the time-dependent receiver operating characteristic curve
OS: Over Survival
95%CI: 95% confidence intervals.
